# A genetic variant in *PIK3R1* is associated with pancreatic cancer survival in the Chinese population

**DOI:** 10.1002/cam4.2228

**Published:** 2019-05-06

**Authors:** Ying Zhu, Juntao Ke, Yajie Gong, Yang Yang, Xiating Peng, Jianbo Tian, Danyi Zou, Nan Yang, Xiaoyang Wang, Shufang Mei, Meilin Rao, Pingting Ying, Yao Deng, Haoxue Wang, Hongli Zhang, Bin Li, Hao Wan, Yue Li, Siyuan Niu, Yimin Cai, Ming Zhang, Zequn Lu, Rong Zhong, Xiaoping Miao, Jiang Chang

**Affiliations:** ^1^ Department of Epidemiology and Biostatistics, Key Laboratory for Environment and Health, School of Public Health Tongji Medical College, Huazhong University of Sciences and Technology Wuhan China

**Keywords:** genetic variant, genome‐wide association study, pancreatic cancer survival, PI3K

## Abstract

Pancreatic cancer is one of the deadliest malignancies with few early detection tests or effective therapies. PI3K‐AKT signaling is recognized to modulate cancer progression. We previously identified that a genetic variant in *PKN1* increased pancreatic cancer risk through the PKN1/FAK/PI3K/AKT pathway. In order to investigate the associations between genetic variations in that pathway and pancreatic cancer prognosis, we conducted a two‐stage survival analysis in a total of 547 Chinese pancreatic cancer patients. Consequently, a variant, rs13167294 A>C in *PIK3R1*, was significantly associated with poor survival in both stages and with hazard ratio being 1.32 (95% CI = 1.13‐1.56, *P* = 0.0007) in the combined analysis. Function annotation and prediction suggested that genetic variants in this locus might affect overall survival of pancreatic cancer patients by regulating *PIK3R1* expression.

## 1 INTRODUCTION

Pancreatic ductal adenocarcinoma (hereafter referred to as pancreatic cancer, PC) is a highly lethal human cancer. In 2018, an estimated 432 243 patients died of the disease, with the fatality rate reaching up to 94.2%.^1^ Due to the lack of early detection methods and effective treatments, the prognosis has been dismal over the past decades. In fact, the survival times vary with each patient and are partly explained by traditional clinical and pathological features.^2^ Moreover, germline variants, ie, single nucleotide polymorphisms (SNP) have been demonstrated associated with overall survival with increasing evidence.^3^, ^4^, ^5^, ^6^, ^7^

PI3K‐AKT signaling is a front and center pathway that is often overactivated in a wide range of tumor types. It triggers a cascade of response, from cell growth to survival and motility, that drive tumor initiation to progression.^8^ Recently, we discovered that a germline variant in *PKN1* gene might perturb the PKN1/FAK/PI3K/AKT pathway and lead to pancreatic tumorigenesis.^9^ Our study bridged the core elements of PI3K‐AKT signaling with PKN1/FAK, and highlighted the role of the germline variant on this pathway played in PC development. PKN1 and FAK had been reported to play a part in cancer progression and metastasis.^10^, ^11^ But the association between genetic variants in this extended pathway and patient survival remained elusive.

Therefore, we hypothesized that genetic variants in the PKN1/FAK/PI3K/AKT pathway were associated with the survival of PC patients, and we tested this hypothesis with a two‐stage survival analysis in a total of 547 subjects.

## 2 MATERIALS AND METHODS

### 2.1 Study subjects

We performed a two‐stage study to search for genetic variants that correlated with PC survival. In the discovery stage, we included 341 patients with complete follow‐up information and genotyped in both of our previous genome‐wide association studies (GWAS) (ChinaPC) 12 and exome‐wide association studies (EWAS).^9^ These subjects were recruited from the Cancer Hospital, Chinese Academy of Medical Sciences, Beijing, between 2000 and 2011. As for the replication stage, 206 PC patients, from Tongji Hospital of Huazhong University of Science and Technology, Wuhan, were enrolled in our study. Characteristics and clinical information including age, gender, smoking and drinking status and tumor stage were obtained from patients’ medical records. Smoking status dichotomized subjects into two categories as we did in former studies 13, 14, 15: (a) Patients who had smoked ≥ 1 cigarette per day and for ≥ 1 year before cancer diagnosis were regarded as smokers (current or former); (b) The rest were nonsmokers. A similar classification went for drinking status: (a) Subjects who drank more than twice a week and for ≥ 1 year were considered as drinkers (current or former); (b) Others were nondrinkers. Tumor stages were harmonized into three categories: (a) local disease amenable to surgical resection; (b) locally advanced disease with extra‐pancreatic extension rendering it unresectable, but without distant metastases; and (c) distant metastatic disease. Survival time was measured from the date of surgery to last follow‐up or death, and the follow‐up information was obtained through telephone calls. Informed consents were obtained from all individuals.

### 2.2 Selection of genes and SNPs

Through literature research, we found a total of 16 genes as follows that are closely related to the PKN1/FAK/PI3K/AKT pathway. Among which, *RAC1*, *RHOA*, *RHOB* and *CASP3* are upstream genes encoding small G proteins or caspase‐3 that activate PKN1.^16^, ^17^, ^18^, ^19^, ^20^ The other 12 genes (*PDK1*, *PKN1*, *FAK*, *PIK3CA*, *PIK3CB*, *PIK3CD*, *PIK3R1*, *PIK3R2*, *PIK3R3*, *AKT1*, *AKT2*, *AKT3*) are members of the PKN1/FAK/PI3K/AKT phosphorylation pathway.^21^, ^22^, ^23^, ^24^ The regulatory relationships are illustrated in Figure S1. We retrieved all of the experimentally genotyped SNPs (minor allele frequency ≥ 0.05) from the GWAS and EWAS dataset within the above genes or their ±10 kilobase flanking regions. All selected SNPs passed the quality control.

### 2.3 Genotyping

Genomic DNA was isolated from peripheral‐blood cells. In the discovery stage, samples were genotyped with the Affymetrix GeneChip Human Mapping 6.0 set and the Illumina HumanExome Beadchip.^9^, ^12^ For the replication stage, four variants were subsequently genotyped using a TaqMan assay on the ABI PRISM 7900 HT platform (Applied Biosystems, Inc).

### 2.4 Function annotation and prediction

All SNPs in linkage disequilibrium (LD) (*r* ^2^ > 0.2, 1000G Phase 1 Asian population) with the tag SNP in a ±200 kb flanking region were selected for function annotation and prediction. RegulomeDB (http://www.regulomedb.org/) and HaploReg v4.1 (https://pubs.broadinstitute.org/mammals/haploreg/haploreg.php), two online function annotation softwares especially for noncoding genetic variants, were used. Those SNPs with RegulomeDB score ≤ 2 (the threshold was set with reference to literature 25, 26), and located in DNase hypersensitive sites or histone modification marker peaks were predicted to be of regulatory function. GTEx eQTL Calculator (https://gtexportal.org/home/testyourown) was used for expression quantitative trait loci analysis. SurvExpress (http://bioinformatica.mty.itesm.mx:8080/Biomatec/SurvivaX.jsp) was used to assess the association between gene expression and survival time.

### 2.5 Statistical analysis

Cox proportional hazards regression was used to measure the effects of candidate SNPs on PC survival, in different genetic models (additive, dominant and recessive) with adjustment for age, gender, smoking and drinking status and stage of disease. Kaplan‐Meier survival estimates were plotted and *P* values were assessed using the log‐rank test. Survival analyses using “coxph” function in “Survival package” were performed with R (3.5.2). For all analyses, *P* < 0.05 was regarded as statistical significance and all tests were two‐sided.

## 3 RESULTS

### 3.1 Characteristics of study subjects

The current study included a discovery dataset of 341 PC patients from our previous GWAS and EWAS studies,^9^, ^12^ and a replication stage of 206 PC patients recruited form Wuhan. The characteristics are shown in Table 1. Demographic characteristics including age, gender, smoking and drinking status showed no significant influence on survival time in two stages and combined analysis. Clinical stage of disease, consistent with general knowledge, was strongly correlated with survival time.

**Table 1:** Selected characteristics of study subjects

	Discovery stage (N = 341)				Replication stage (N = 206)				Combined stage (N = 547)			
	No. of Patients (%)	No. of Death (%)	MST (months)	*P* a	No. of Patients (%)	No. of Death (%)	MST (months)	*P* a	No. of Patients (%)	No.of Death (%)	MST (months)	*P* a
Age at diagnosis				0.4360				0.3489				0.3718
≤60	153 (44.87)	136 (88.89)	7.4		93 (45.1)	67 (72.0)	9.2		335 (61.2)	279 (83.3)	8.2	
>60	188 (55.13)	176 (93.62)	7.2		113 (54.9)	88 (77.9)	8.3		212 (38.8)	188 (88.7)	7.6	
Gender				0.3208				0.9921				0.1731
Male	202 (59.24)	180 (89.11)	7.4		133 (64.6)	99 (74.4)	8.6		246 (45.0)	203 (82.5)	7.9	
Female	139 (40.76)	132 (94.96)	7.2		73 (35.4)	56 (76.7)	9.8		301 (55.0)	264 (87.7)	7.8	
Smoking status				0.5021				0.4905				0.3473
Yes	74 (21.70)	68 (91.89)	6.9		52 (25.2)	43 (82.7)	8.6		421 (77.0)	356 (84.6)	7.3	
No	267 (78.30)	244 (91.39)	7.6		154 (74.8)	112 (72.7)	8.9		126 (23.0)	111 (88.1)	8.0	
Drinking status				0.9305				0.9296				0.9963
Yes	62 (18.18)	56 (90.32)	6.8		38 (18.4)	30 (78.9)	10.3		447 (81.7)	381 (85.2)	8.1	
No	279 (81.82)	256 (91.76)	7.4		168 (81.6)	125 (74.4)	8.5		100 (18.3)	86 (86.0)	7.8	
Clinical stage				1.98E‐08				3.37E‐15				9.56E‐21
Local	72 (21.11)	61 (84.72)	9.3		54 (26.2)	29 (53.7)	18.8		126 (23.0)	90 (71.4)	13.9	
Locally advanced	151 (44.28)	133 (88.08)	8.9		87 (42.2)	70 (80.5)	9.2		238 (43.5)	203 (85.3)	9.1	
Metastatic	118 (34.60)	118 (100.00)	5.1		65 (31.6)	56 (86.2)	4.2		183 (33.5)	174 (95.1)	4.8	

Abbreviation: MST, median survival time.

a Calculated using the log‐rank test.

### 3.2 Multivariate analyses of associations between SNPs and patient survival

**Figure 1:**
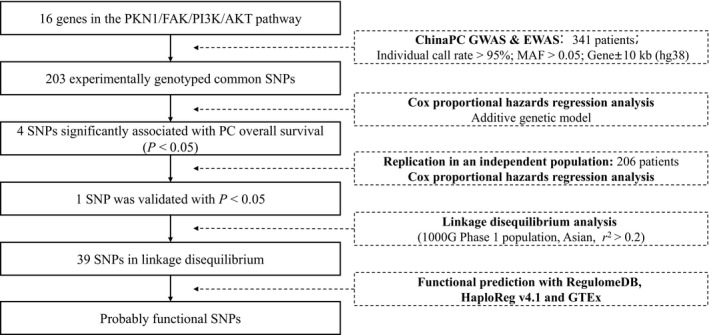
Research workflow for SNPs in the PKN1/FAK/PI3K/AKT pathway genes. EWAS, exome‐wide association study; GWAS, genome‐wide association study; MAF, minor allele frequency; SNP, single nucleotide polymorphism

**Figure 2:**
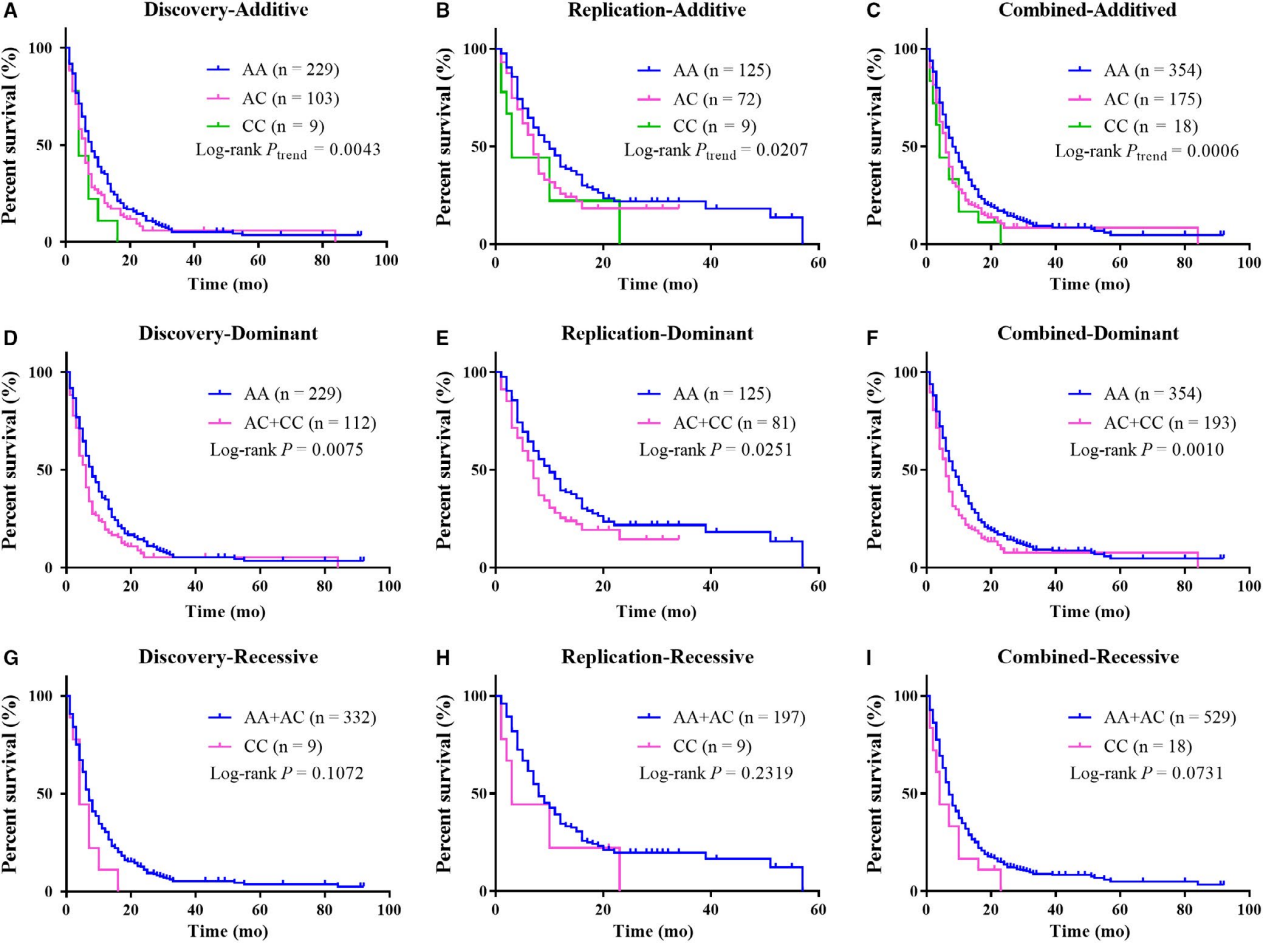
Kaplan‐Meier curves of survival in pancreatic cancer patients by rs13167294 genotypes, in different stages, assuming (a‐c) additive, (d‐f) dominant and (g‐i) recessive models

The design flowchart of the present study was illustrated in Figure 1. At first, a total of 203 genotyped common SNPs (minor allele frequency > 0.05) in GWAS (ChinaPC) and EWAS were selected for which were located in the 16 PKN1/FAK/PI3K/AKT pathway genes or their 10 kb flanking regions. Next, the associations between the 203 SNPs with PC survival were assessed with Cox proportional hazards regression under an additive model adjusting for age, gender, smoking and drinking status and clinical stage. Among these SNPs, four were significantly associated with patient survival and passed for validation in an independent population (Table 2). As a result, only one SNP, rs13167294 in *PIK3R1*, was successfully replicated and remained significantly associated with survival time in the combined analysis. The minor C‐allele conferred poorer prognosis for patients, and the per‐allele hazard ratio for which was 1.32 (95% CI = 1.13‐1.56, *P* = 0.0007) in the combined analysis. rs13167294 A>C was also correlated with decreased survival under a dominant model in discovery, replication and combined stages (Table 3). Further stratification analyses showed that the significant correlation was particularly in patients with metastatic stage of disease. As for patients in locally advanced stage, the minor C‐allele also indicated worse survival than the reference, but the association of SNP and survival was not statistically significant possibly due to the relatively small sample size. Further research with larger sample size is still needed to draw firm conclusion about the effects of rs13147249 in different stages (Table S1). Kaplan‐Meier curves illustrated significant associations between this variation and survival especially in additive and dominant models (Figure 2).

**Table 2:** Analysis of the four SNPs that passed the screen of discovery stage

SNP	Allele^a^	Gene	Discovery stage		Replication stage		Combined stage	
			HR (95% CI)	*P* b	HR (95% CI)	*P* b	HR (95% CI)	*P* b
rs13167294	A>C	*PIK3R1*	1.31 (1.07‐1.61)	0.0104	1.45 (1.10‐1.91)	0.0076	1.32 (1.13‐1.56)	0.0007
rs7839119	C>A	*FAK*	0.76 (0.62‐0.95)	0.0138	1.02 (0.76‐1.36)	0.8905	0.83 (0.70‐0.99)	0.0369
rs4983387	G>A	*ZBTB42* c	1.21 (1.02‐1.42)	0.0252	1.13 (0.87‐1.46)	0.3525	1.18 (1.03‐1.35)	0.0192
rs342105	T>C	*RHOB*	1.57 (1.01‐2.44)	0.0471	0.94 (0.57‐1.55)	0.8068	1.13 (0.82‐1.56)	0.4703

Abbreviations: HR, hazard ratio; SNP, single nucleotide polymorphism.

a Reference allele/effect allele.

b Calculated using Cox regression in an additive genetic model adjusting for age, gender, smoking, drinking status and clinical stage.

c This SNP locates in the exon of *ZBTB42*, and 6.1kb upstream of *AKT1*.

**Table 3:** Association between rs13167294 and overall survival in pancreatic cancer patients

	Discovery stage				Replication stage				Combined stage			
	No. (%)	MST	HR (95% CI)	*P* a	No. (%)	MST	HR (95% CI)	*P* a	No. (%)	MST	HR (95% CI)	*P* a
*PIK3R1* rs13167294												
AA	229 (67.2)	8.32	1.00 (Reference)		125 (60.7)	10.62	1.00 (Reference)		354 (64.7)	8.95	1.00 (Reference)	
AC	103 (30.2)	6.27	1.34 (1.05‐1.72)	0.0192	72 (35.0)	7.60	1.44 (1.02‐2.04)	0.0373	175 (32.0)	6.69	1.36 (1.11‐1.65)	0.0026
CC	9 (2.6)	4.83	1.57 (0.80‐3.07)	0.1913	9 (4.4)	3.75	2.13 (1.02‐4.43)	0.0434	18 (3.3)	4.67	1.63 (1.00‐2.67)	0.0512
Additive model			1.31 (1.07‐1.61)	0.0104			1.45 (1.10‐1.91)	0.0076			1.32 (1.13‐1.56)	0.0007
Dominant model			1.36 (1.07‐1.73)	0.0117			1.51 (1.08‐2.10)	0.0151			1.38 (1.14‐1.67)	0.0010
Recessive model			1.45 (0.74‐2.82)	0.2812			1.88 (0.92‐3.87)	0.0859			1.48 (0.91‐2.42)	0.1119

Abbreviation: HR, hazard ratio; MST, median survival time.

a Calculated using Cox regression adjusting for age, gender, smoking, drinking status and clinical stage.

### 3.3 Functional variants potentially affect PC prognosis by regulating *PIK3R1* expression

In order to further seek functional variants related to the identified locus, we retrieved 39 SNPs in LD (*r* ^2^ > 0.2, 1000G Phase 1 Asian population) with rs13167294. These SNPs were all noncoding variants (38 intronic and one in the 5′UTR) of *PIK3R1*. So we utilized RegulomeDB and HaploReg v4.1 to assess whether each one of the 39 SNPs was functional. Those SNPs with RegulomeDB score ≤ 2, and located in DNase hypersensitive sites or histone modification marker peaks (ie rs1819986 and rs6876003) were predicted to be of regulatory function probably by regulating the expression of *PIK3R1* (Table S2). Utilizing the GTEx eQTL Calculator, we found that the associations between genotypes and *PIK3R1* expressions in pancreas tissue were significant for six SNPs (ie rs6876003, rs6890202, rs1819987, rs6894871, rs6861401 and rs1010793). Taking account of results from the above estimates, the variant rs6876003 was most likely functional by affecting *PIK3R1* expression (Table S2). Next, we took advantage of SurvExpress to assess the association between *PIK3R1* expressions and overall survival in PC patients. The higher expression of *PIK3R1* suggested worse survival in two public datasets: Stratford Yeh Pancreatic GSE21501 and Pancreatic Cancer‐AU (PACA‐AU‐ICGC‐June 2016, Australian Pancreatic Cancer Genome Initiative) (Figure S2). Taken together, genetic variants such as rs6876003 might influence PC prognosis by regulating *PIK3R1* mRNA expression levels.

## 4 DISCUSSION

In the present study, we performed a two‐stage survival analysis to specifically estimate the association between overall survival in PC patients and genetic variants of the 16 genes belonging to the PKN1/FAK/PI3K/AKT pathway. In the discovery stage, we screened 203 corresponding variants and found four of prognostic significance. Among which was only one variant, rs13167294 in *PIK3R1*, successfully validated in an independent population in the replication stage. For the combined analysis of 547 patients in total, rs13167294 remained significantly correlated with PC survival under both additive and dominant genetic model. Subsequent in silico functional prediction suggested variants in the locus tagged by rs13167294 might affect survival by regulation of *PIK3R1* expression.

In the discovery stage, we found four potentially associated SNPs with the lowest *P* value of 0.0104. Partly due to the relatively small sample size, the *P* values would be no longer significant after multiple comparisons correction. In fact, the aim of the discovery stage was to screen out the candidate SNPs as completely as possible. Therefore, we did not adopt corrected *P* values for multiple comparisons, in case the actual associated SNPs were excluded by the strict threshold. As for the replication and combined stages, we took Bonferroni correction for the four candidate SNPs, and found that rs13167249 was significantly associated with survival even at the corrected threshold of *P* < 0.0125.

It is well recognized that the PI3K‐AKT signaling plays critical roles in metastasis of cancers including PC.^27^, ^28^, ^29^, ^30^ It promotes cellular events such as invasion, migration and epithelial‐mesenchymal transition via regulation of different transcription factors.^31^, ^32^, ^33^ *PIK3R1*, which encodes the 85 kD regulatory subunit of PI3K, functions primarily as a regulator of the p110α catalytic product. Its mutations and abnormal expression were reported to impair PI3K function, and further cause disease.^34^, ^35^, ^36^, ^37^ An integrative survival‐based molecular profiling of human pancreatic cancer had identified *PIK3R1* expression as a putative clinical biomarker for the disease outcome.^38^ But little was known about the cis‐regulatory element of this gene. Our current study identified that several genetic variants in *PIK3R1* might regulate the expression of this gene. Take the most likely functional variant rs6876003 for instance, this intronic variant locates in a DNase I hypersensitive site, where histone modification predicts an active enhancer function. It potentially affects TF binding since it resides in some motifs, thus supporting its regulatory function. In particular, the genotype‐phenotype correlation demonstrated the modulation of *PIK3R1* expression levels by rs6876003, suggesting that the findings were biologically plausible that this variant played a role in PI3K signaling and even PC prognosis. It should be admitted that the association between PC survival and rs6876003 were not completely represented by the result of the tagSNP rs13167294 since the LD relationship is not strong enough (*r* ^2^ = 0.32). However, the reason for choosing a weak LD threshold is to find, as much as possible, potentially functional SNP for further research. As for the eQTL analysis, we only found available data from pancreatic normal tissues in the public databases. Genotype‐phenotype data from cancer tissues was lacking, which was a limitation of the present study. It is also worth noting that other variants in the LD list might also be functional such as those with RegulomeDB score > 2. In fact, the bioinformatics tools just offered us some clues, and biological experiment is needed to prove them in the future.

In summary, we identified a variant in *PIK3R1* associated with PC outcome. Further studies of larger sample size are required to validate our findings.

## CONFLICTS OF INTEREST

The authors declare no conflict of interest.
