# Supplementary figures and images for: A genetic variant in PIK3R1 is associated with pancreatic cancer survival in the Chinese population
Source: Cancer Med. 2019 May 6;8(7):3575–82. doi: 10.1002/cam4.2228 (PMC6601582; doi:10.1002/cam4.2228)

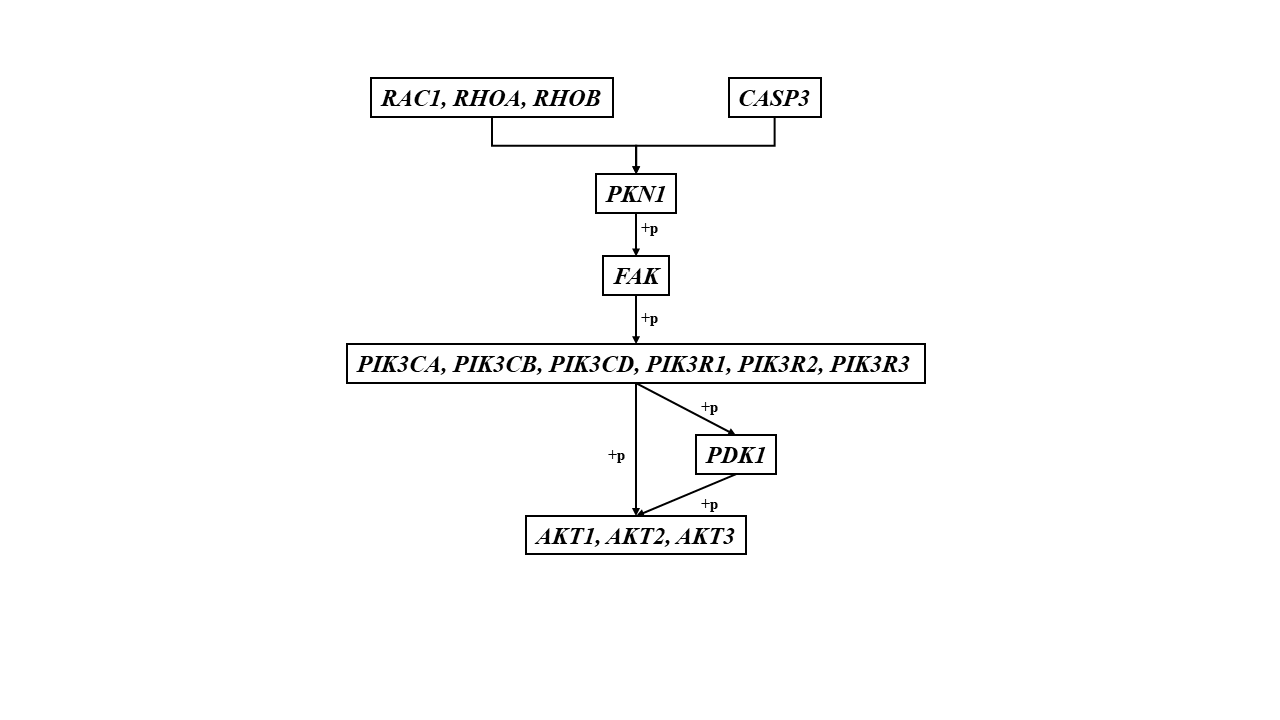

Supplement: Supplementary file 1 [file CAM4-8-3575-s001.tif]

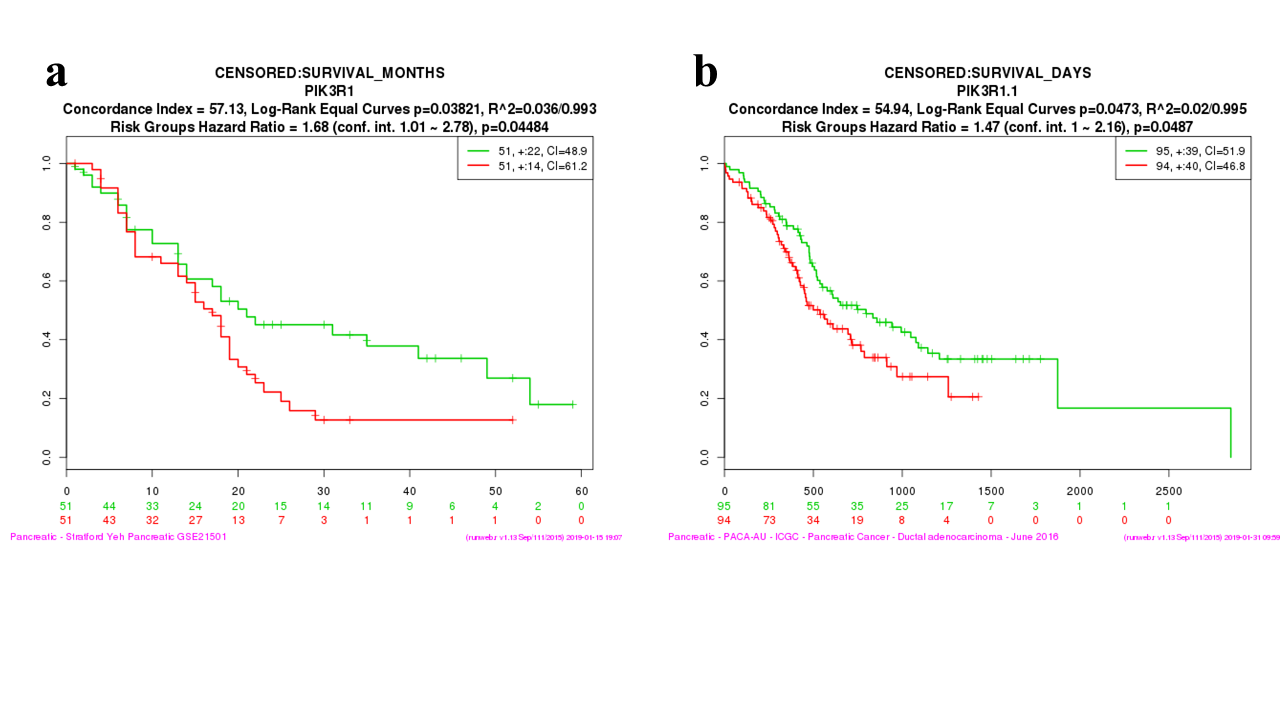

Supplement: Supplementary file 2 [file CAM4-8-3575-s002.tif]
