# Supplementary material for: A genetic variant in PIK3R1 is associated with pancreatic cancer survival in the Chinese population
Source: Cancer Med. 2019 May 6;8(7):3575–82. doi: 10.1002/cam4.2228 (PMC6601582; doi:10.1002/cam4.2228)
Supplement: Supplementary file 3 [file CAM4-8-3575-s003.docx]

**Supplementary Table 1. Stratification analyses of rs13167294 based on clinical disease stage in the combined stage.**

|  | **No. (%)** | **MST** | **HR (95% *CI*)** | ***P*^†^** |
| --- | --- | --- | --- | --- |
| **Local Stage** |  |  |  |  |
| AA | 86 (68.3) | 14.75 | 1.00 (Reference) |  |
| AC | 37 (29.4) | 8.62 | 1.28 (0.80-2.04) | 0.3083 |
| CC | 3 (2.4) | 23.25 | 0.58 (0.14-2.47) | 0.4629 |
| Additive model |  |  | 1.06 (0.72-1.56) | 0.7665 |
| Dominant model |  |  | 1.18 (0.75-1.86) | 0.4785 |
| **Locally Advanced Stage** |  |  |  |  |
| AA | 158 (66.4) | 10.06 | 1.00 (Reference) |  |
| AC | 75 (31.5) | 8.08 | 1.21 (0.90-1.64) | 0.2103 |
| CC | 5 (2.1) | 10.25 | 1.27 (0.52-3.14) | 0.5983 |
| Additive model |  |  | 1.19 (0.92-1.54) | 0.1965 |
| Dominant model |  |  | 1.22 (0.91-1.63) | 0.1903 |
| **Metastatic Stage** |  |  |  |  |
| AA | 110 (60.1) | 5.33 | 1.00 (Reference) |  |
| AC | 63 (34.4) | 4.23 | 1.53 (1.10-2.14) | 0.0118 |
| CC | 10 (5.5) | 3.00 | 2.08 (1.07-4.05) | 0.0313 |
| Additive model |  |  | 1.49 (1.15-1.92) | 0.0030 |
| Dominant model |  |  | 1.59 (1.16-2.19) | 0.0045 |

^†^Calculated using Cox regression adjusting for age, gender, smoking and drinking status.

.Abbreviation: HR, Hazard Ratio; CI, confidence interval; MST, median survival time

**Supplementary Table 2. Function annotation of SNPs in the locus tagged by rs13168294.**

| **Related SNP** | **Position (hg38)** | **LD (r^2^)**^†^ | **Gene** | **RegulomeDB score** | **Haploreg v4.1** | | | | | | | **GTEx *P*_eQTL-Pancreas_** |
| --- | --- | --- | --- | --- | --- | --- | --- | --- | --- | --- | --- | --- |
|  |  |  |  |  | **Promoter histone marks** | **Enhancer histone marks** | **DNAse** | **Proteins bound** | **Motifs changed** | **GRASP QTL hits** | **dbSNP func annot** |  |
| rs1819986 | Chr5 : 68271248 | 0.79 | *PIK3R1* | 2b | BRST, BLD | 12 tissues | HRT | 5 bound proteins | 8 altered motifs |  | intronic | 0.1 |
| rs6876003 | Chr5 : 68275585 | 0.32 | *PIK3R1* | 2b |  | 4 tissues | 5 tissues |  | ATF3,DMRT2,ERalpha-a |  | intronic | 0.034 |
| rs972634 | Chr5 : 68255643 | 1.00 | *PIK3R1* | 3a | 7 tissues | 16 tissues | 12 tissues |  | 12 altered motifs |  | intronic | 0.058 |
| rs10940160 | Chr5 : 68267399 | 0.33 | *PIK3R1* | 3a |  |  | HRT,LNG |  | 5 altered motifs |  | intronic | 0.14 |
| rs200396786 | Chr5 : 68271584 | 0.79 | *PIK3R1* | 3a | FAT | 8 tissues |  |  | 13 altered motifs |  | intronic | NA |
| rs11360241 | Chr5 : 68271585 | 0.79 | *PIK3R1* | 3a | FAT | 8 tissues |  |  | 14 altered motifs |  | intronic | 0.11 |
| rs11746842 | Chr5 : 68272376 | 0.79 | *PIK3R1* | 3a |  | 7 tissues | MUS,OVRY | CTCF,RAD21 | 10 altered motifs |  | intronic | 0.34 |
| rs10065413 | Chr5 : 68272701 | 0.79 | *PIK3R1* | 3a |  |  | BLD |  | 6 altered motifs |  | intronic | 0.32 |
| rs6890202 | Chr5 : 68275134 | 0.33 | *PIK3R1* | 3a | BLD | 11 tissues | BLD,BLD,SKIN | EBF1,NFKB | RP58,TAL1 |  | intronic | 0.019 |
| rs7709991 | Chr5 : 68257141 | 1.00 | *PIK3R1* | 4 | BLD, GI | 17 tissues | BLD,HRT | ZNF263 |  |  | intronic | 0.066 |
| rs2161120 | Chr5 : 68268368 | 0.33 | *PIK3R1* | 4 | BLD, GI | 14 tissues | 4 tissues |  | DMRT7 |  | intronic | 0.086 |
| rs1819987 | Chr5 : 68271225 | 0.33 | *PIK3R1* | 4 | BRST, BLD | 12 tissues | HRT | 4 bound proteins | GR |  | intronic | 0.019 |
| rs3730082 | Chr5 : 68274310 | 0.79 | *PIK3R1* | 4 |  | 10 tissues | BRST,BRST |  |  |  | intronic | 0.1 |
| rs6894871 | Chr5 : 68275442 | 0.33 | *PIK3R1* | 4 | BLD | 6 tissues | 5 tissues |  | GR |  | intronic | 0.019 |
| rs138814985 | Chr5 : 68288512 | 0.48 | *PIK3R1* | 4 | 23 tissues |  | 38 tissues | 8 bound proteins | TATA |  | 5'-UTR | 0.93 |
| rs4122269 | Chr5 : 68249796 | 0.91 | *PIK3R1* | 5 | SKIN, GI | 17 tissues | ESDR |  | Elf5,Nrf-2,PU.1 | 3 hits | intronic | 0.76 |
| rs1823023 | Chr5 : 68250578 | 0.38 | *PIK3R1* | 5 | 5 tissues | 21 tissues | 5 tissues |  |  |  | intronic | 0.075 |
| rs1946208 | Chr5 : 68262593 | 0.32 | *PIK3R1* | 5 |  | 10 tissues | 4 tissues |  | 5 altered motifs |  | intronic | NA |
| rs16897561 | Chr5 : 68264888 | 0.81 | *PIK3R1* | 5 | BLD, HRT | 20 tissues | BLD,HRT,GI |  | Osf2 | 1 hit | intronic | 0.1 |
| rs12697060 | Chr5 : 68268104 | 0.80 | *PIK3R1* | 5 | BLD, GI | 12 tissues | LNG |  | 14 altered motifs |  | intronic | 0.32 |
| rs2112208 | Chr5 : 68268499 | 0.80 | *PIK3R1* | 5 | 5 tissues | 15 tissues | BLD,HRT,BLD |  | 4 altered motifs |  | intronic | 0.43 |
| rs16897570 | Chr5 : 68268891 | 0.80 | *PIK3R1* | 5 | 8 tissues | 17 tissues | 6 tissues |  | EBF |  | intronic | 0.26 |
| rs2302975 | Chr5 : 68273651 | 0.33 | *PIK3R1* | 5 |  | 10 tissues | HRT,MUS,MUS |  |  | 1 hit | intronic | 0.081 |
| rs7735204 | Chr5 : 68276339 | 0.79 | *PIK3R1* | 5 | BLD | 6 tissues |  |  | DMRT1,STAT |  | intronic | 0.077 |
| rs6860081 | Chr5 : 68276543 | 0.79 | *PIK3R1* | 5 |  | 10 tissues |  |  | 6 altered motifs |  | intronic | 0.077 |
| rs6861401 | Chr5 : 68277065 | 0.32 | *PIK3R1* | 5 |  | 10 tissues |  |  | 9 altered motifs |  | intronic | 0.019 |
| rs200662550 | Chr5 : 68241379 | 0.54 | *PIK3R1* | 6 | BLD | 12 tissues |  |  | 14 altered motifs |  | intronic | NA |
| rs1946207 | Chr5 : 68262592 | 0.30 | *PIK3R1* | 6 |  | 10 tissues | 4 tissues |  | Nanog,Pou5f1 |  | intronic | NA |
| rs55710167 | Chr5 : 68265115 | 0.79 | *PIK3R1* | 6 | LIV | 20 tissues |  |  | GR,NRSF,Pbx3 |  | intronic | 0.1 |
| rs201947394 | Chr5 : 68267768 | 0.69 | *PIK3R1* | 6 | BLD, GI, SKIN | 13 tissues | LNG |  | 5 altered motifs |  | intronic | NA |
| rs67623950 | Chr5 : 68267769 | 0.72 | *PIK3R1* | 6 | BLD, GI, SKIN | 13 tissues | LNG |  | 7 altered motifs |  | intronic | NA |
| rs10940161 | Chr5 : 68272157 | 0.78 | *PIK3R1* | 6 |  |  | OVRY |  | 26 altered motifs |  | intronic | 0.58 |
| rs3730088 | Chr5 : 68292172 | 0.37 | *PIK3R1* | 6 | 7 tissues | 13 tissues |  |  | 14 altered motifs |  | intronic | 0.96 |
| **rs13167294^‡^** | **Chr5 : 68259752** |  | ***PIK3R1*** | **7** |  | **11 tissues** | **HRT** |  |  |  | **intronic** | **0.24** |
| rs56352616 | Chr5 : 68265168 | 0.81 | *PIK3R1* | 7 | LIV | 20 tissues | BLD,HRT |  | Mtf1,NRSF,Smad |  | intronic | 0.14 |
| rs10072475 | Chr5 : 68272900 | 0.79 | *PIK3R1* | 7 |  |  |  |  | HNF1,PLZF |  | intronic | 0.78 |
| rs7716675 | Chr5 : 68275943 | 0.79 | *PIK3R1* | 7 |  | 4 tissues | BLD,BLD |  | Hbp1 |  | intronic | 0.077 |
| rs1010793 | Chr5 : 68276040 | 0.32 | *PIK3R1* | 7 |  |  |  |  | NF-Y,Nkx3 |  | intronic | 0.019 |
| rs831124 | Chr5 : 68278922 | 0.22 | *PIK3R1* | 7 |  | 4 tissues |  |  | RFX5 |  | intronic | 0.16 |

^†^ r^2^ measuring the linkage disequilibrium between SNPs and rs13167294 was calculated with 1000G Phase 1 Asian population.

**^‡^** Tag SNP

Abbreviations: SNP, single-nucleotide polymorphism; Chr, chromosome; dbSNP func annot, dbSNP function annotation.
